# Different Mechanisms Must Be Considered to Explain the Increase in Hippocampal Neural Precursor Cell Proliferation by Physical Activity

**DOI:** 10.3389/fnins.2016.00362

**Published:** 2016-08-03

**Authors:** Rupert W. Overall, Tara L. Walker, Tim J. Fischer, Moritz D. Brandt, Gerd Kempermann

**Affiliations:** ^1^Genomics of Regeneration, Center for Regenerative Therapies Dresden (CRTD), Technische Universität DresdenDresden, Germany; ^2^Genomics of Regeneration, German Center for Neurodegenerative Diseases (DZNE) DresdenDresden, Germany; ^3^Division of Neurodegenerative Diseases, Department of Neurology, Technische Universität DresdenDresden, Germany

**Keywords:** adult neurogenesis, dentate gyrus, physical exercise, running, cell cycle, S phase

## Abstract

The number of proliferating neural precursor cells in the adult hippocampus is strongly increased by physical activity. The mechanisms through which this behavioral stimulus induces cell proliferation, however, are not yet understood. In fact, even the mode of proliferation of the stem and progenitor cells is not exactly known. Evidence exists for several mechanisms including cell cycle shortening, reduced cell death and stem cell recruitment, but as yet no model can account for all observations. An appreciation of how the cells proliferate, however, is crucial to our ability to model the neurogenic process and predict its behavior in response to pro-neurogenic stimuli. In a recent study, we addressed modulation of the cell cycle length as one possible mode of regulation of precursor cell proliferation in running mice. Our results indicated that the observed increase in number of proliferating cells could not be explained through a shortening of the cell cycle. We must therefore consider other mechanisms by which physical activity leads to enhanced precursor cell proliferation. Here we review the evidence for and against several different hypotheses and discuss the implications for future research in the field.

## Introduction

**Figure 1:**
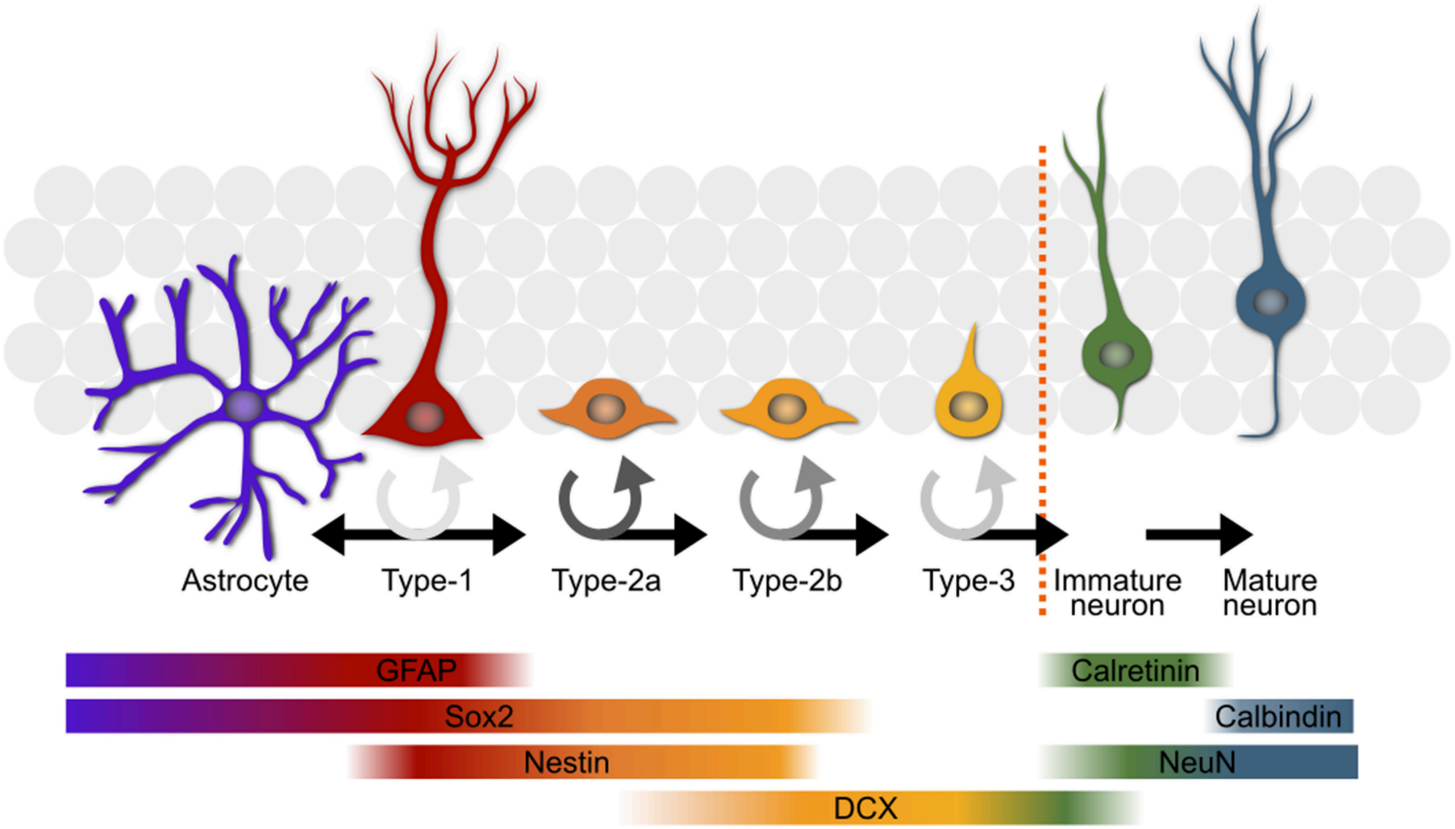
**An overview of the most commonly used model of adult hippocampal neurogenesis**. The type-1 stem cells (second from left in red) either directly produce astrocytes (far left in purple) or give rise to progenitor cells which progress through several stages before becoming mature neurons or being eliminated through apoptosis. The color of the circular arrows represents average proliferation rate of the cells at different stages; darker shades equate to a more proliferative population. Some of the key markers used in the study of adult neurogenesis are shown with their expression profiles below. GFAP, glial fibrillary acidic protein; DCX, doublecortin.

A **stem cell niche** exists in the subgranular zone of the hippocampus of adult mice—as well as most other mammals, including humans. The pool of stem cells residing in this niche produces new adult-born neurons and astrocytes throughout the lifetime of the organism. A **stem cell** passes through several defined stages (Kempermann et al., 2004; Overall et al., 2012; Figure 1), each of which appears to be regulated to a certain degree independently (Kronenberg et al., 2003), on the path to becoming a functionally mature astrocyte or neuron. The stem cells, or type-1 cells (Kempermann et al., 2004), are radial glia-like cells which divide either symmetrically (Bonaguidi et al., 2011), or asymmetrically to yield a daughter astrocyte (Bonaguidi et al., 2011; Encinas et al., 2011; Gebara et al., 2016) or type-2 neuronal **progenitor cell** (Bonaguidi et al., 2011; Encinas et al., 2011; Gebara et al., 2016). The type-2 stage has been subdivided into two sub-stages, type-2a and -2b, based on the expression of glia-like vs. early neuronal markers (Hodge et al., 2008). The type-2a cells are highly prolific and appear to be the major responders to the running stimulus (Kronenberg et al., 2003). Type-2b is defined by the onset of doublecortin expression, a marker associated with the migrating neuroblast stage in adult-born olfactory bulb neuron maturation (Brown et al., 2003). A final proliferative stage, type-3, precedes exit from the cell cycle and the transient expression of calretinin (CR; Brandt et al., 2003). These new-born immature neurons then experience a phase lasting up to 7 weeks (Kempermann et al., 2004) during which they are selected for further differentiation into a functionally mature neuron or elimination, presumably through apoptosis (Young et al., 1999). The signals promoting long-term survival of the new neurons are still not known but likely involve synaptic activation from the surrounding established granule cell network. The molecular mechanisms governing proliferation of the **precursor cell** population and their survival as new neurons seem to be distinct (Kempermann et al., 2006) with the rate of proliferation being more strongly influenced by physical activity in comparison to the predominantly pro-survival effect of environmental enrichment (Kempermann et al., 1997; van Praag et al., 1999; Fabel et al., 2009).

KEY CONCEPT 1. Stem cell niche The microenvironment in which stem cells reside. In adult hippocampal neurogenesis, this is the subgranular zone at the interface between the granule cell layer of the dentate gyrus and the hilus. A range of different cell types make contact with the stem cells and are involved in cell signaling crosstalk.

KEY CONCEPT 2. Stem cell A stem cell is defined by the properties of unlimited self-renewal and the ability to differentiate into one or more mature cell types. The neural stem cells in the adult hippocampus (“type-1” cells) have the potential to generate new astrocytes, oligodendrocytes and neurons. They can also enter into a quiescent state for some time before being activated to re-enter the cell cycle.

KEY CONCEPT 3. Progenitor cell A progenitor cell is the progeny of an asymmetric stem cell division and is defined by the commitment to follow a particular lineage. These cells exhibit limited self-renewal. In adult hippocampal neurogenesis these include the rapidly amplifying type-2a cells as well as the later type-2a and type-3 populations.

KEY CONCEPT 4. Precursor cell Precursor cell is used here as a convenience term to encompass both the stem cells and the progenitor cells.

Although the rate of stem and precursor cell proliferation is strongly influenced by the genetic background of the animal (Kempermann et al., 1997, 2006; van Praag et al., 1999; Kempermann and Gage, 2002a,b; Overall et al., 2013), the remarkable feature of adult hippocampal neurogenesis is its plasticity in response to the environment. In particular, physical activity (typically represented by wheel running in experimental rats and mice) leads to a robust increase in the number of proliferating precursor cells (van Praag et al., 1999; Kronenberg et al., 2003, 2006; Lugert et al., 2010; Overall et al., 2013; Farioli-Vecchioli et al., 2014; Fischer et al., 2014). Wheel running typically results in about a 50% increase in proliferating cells, although this value is highly dependent on the amount of time spent running and the method used for detecting proliferating cells. The dynamics of precursor cell proliferation in response to running are not linear, with a steep rise in the number of proliferating cells reaching a plateau around 10–14 days (Kronenberg et al., 2003, 2006) before declining to baseline after about 4–6 weeks (Kronenberg et al., 2006; Overall et al., 2013). The proliferating cells include several distinct populations—the type-1 stem cells (which themselves fall into a number of morphologically and transcriptionally distinct subpopulations; Lugert et al., 2010; Bonaguidi et al., 2011; Encinas et al., 2011; Shin et al., 2015; Gebara et al., 2016), type-2a, type-2b and type-3—all of which may have different cell cycle lengths (Brandt et al., 2012; Farioli-Vecchioli et al., 2014) and may divide symmetrically or asymmetrically (Lugert et al., 2010; Bonaguidi et al., 2011; Encinas et al., 2011; Gebara et al., 2016). The standard model (Kempermann et al., 2004) is not explicit about whether the cell stage transitions might be the result of cell division or rather a gradual maturation. When the numbers of each cell population are compared in pulse-chase labeling experiments, the results usually do not correspond to a doubling in the next stage (as expected for symmetric division) or equal numbers in two consecutive stages (as expected for asymmetric division) or a shift from one stage to another (as should be seen if the two stages are different maturation states of the same cell population). It may be that combinations of these processes are occurring simultaneously. This question is nearing resolution for the type-1 cells which appear to be capable of a mixture of symmetric and asymmetric divisions to yield new type-1 cells, early progenitors and astrocytes (Lugert et al., 2010; Bonaguidi et al., 2011; Encinas et al., 2011; Gebara et al., 2016). It also appears to be the case that the post-mitotic calretinin-expressing cells are the result of a terminal division (Brandt et al., 2003; see discussion below) although the identity of the parent cell type, or types, for this division has not yet been established. That all of these cells are dividing or undergoing differentiation asynchronously adds a further layer of complexity. Furthermore, the different techniques used to measure proliferation each come with methodological quirks which can often significantly alter the interpretation of results (see the Section “Open Questions” below). Finally, the parameters for most of these factors have not yet been reliably experimentally determined, making the construction of a useful model seemingly intractable.

The result of this complexity is that, despite much work, the cellular mechanism by which physical activity causes increased precursor cell proliferation has still not been established. It is, however, critical for a functional understanding of adult neurogenesis that we have an appreciation of how the different cells that are involved in the process of adult neurogenesis and its activity-dependent regulation proliferate. It is clear that the number of cycling cells increases; but the signals induced by physical activity mediating this increase, and the cells on which these signals act, are still largely unknown. At a more general level, the cellular mechanisms by which proliferation is regulated—such as cell cycle dynamics, cell death, mode of division or fate choice—are also disputed. It will be necessary to establish a comprehensive mechanistic framework before more detailed and dynamic models can be attempted.

This review focuses on two papers which have addressed modulation of cell cycle length as a potential mechanism behind running-induced increases in proliferation. The two studies differed in their conclusions—likely due to our lack of understanding of the complex dynamics of the running effect. We further discuss the need for other mechanisms to fully explain the now extensive literature in the field. Much of the published data does not fit well to existing models and quantitative (and even qualitative) discrepancies abound; as discussed in the following sections. We suggest that improved models are required to account for the existing data and that such models will have to be instructive on the fundamental mechanisms involved in adult neural stem cell proliferation.

## Cell cycle acceleration is not sufficient to explain the running-induced increase in proliferation

One mechanism that has been proposed to explain the increased proliferation after physical exercise is an acceleration of the cell cycle. This idea is based on a long history of studying cell cycle kinetics in development when it was discovered early on that cells can alter the length of their mitotic cycle and that this can be associated with a switch between proliferation and differentiation. Indeed, experimentally lengthening neural precursor cell cycle can induce differentiation (Calegari and Huttner, 2003) and shortening the cell cycle can also hold cells back in a proliferative state both during development (Lange et al., 2009) and in the adult brain (Artegiani et al., 2011). Modification of the cell cycle length has thus presented an intriguing potential mechanism for the increased proliferation of hippocampal precursor cells observed in response to physical activity. This hypothesis has been tested recently in two independent studies which differed in their conclusions. The first, by Farioli-Vecchioli and colleagues (Farioli-Vecchioli et al., 2014), reported that in wild-type mice, running induces a shortening of both the **S phase** (T_S_) and the total cell cycle length (T_C_) and that this occurs specifically in NeuroD1^+^ (type-2/-3) **progenitor cells** but not GFAP^+^/Sox2^+^ (type-1) stem cells in wild-type animals. They proposed a model in which running induces a shortening of the cell cycle and transient expansion of the NeuroD1^+^ type-2b–3 population—returning to baseline levels after withdrawal of the running stimulus as these cells differentiate. While they showed a 23% increase in the number of BrdU^+^ cells (2 h after injection) and an increase of 30% in expression of the endogenous marker Ki67, there was no change observed in numbers of either radial glia-like type-1 cells (Nestin^+^/GFAP^+^) or mitotic radial glia-like stem cells (Nestin^+^/GFAP^+^/Ki67^+^). They thus concluded that their data are not consistent with a model in which running induces recruitment of quiescent stem cells.

KEY CONCEPT 5. S phase The phase of the cell cycle in which new DNA is synthesized while the chromosomes are copied in readiness for the coming cell division. During this process, deoxynucleoside analogs such as BrdU can be incorporated to mark the dividing cell.

A similar study from our own group, however, did not find a significant shortening of the length of the cell cycle or S phase of hippocampal precursor cells following physical activity. This was despite a robust 38% increase in the number of proliferating cells. In fact, we calculated that the small differences observed (a shortening of T_S_ by 0.27 h and T_C_ by 1.81 h compared to the differences in T_S_ of 2.65 and T_C_ of 2.88 reported by Farioli-Vecchioli et al.), even if statistically significant and assuming extreme parameters, could still not explain the increase in proliferation that was observed (Fischer et al., 2014).

Both studies employed a similar methodology using double-labeling with the mutually distinguishable thymidine analogs 5-chloro-2′-deoxyuridine (CldU) and 5-iodo-2′-deoxyuridine (IdU) to calculate the lengths of S phase and the total cell cycle (Shibui et al., 1989; Hayes and Nowakowski, 2002; Brandt et al., 2012). Estimates of the steady-state cell cycle length were similar in both cases (see Table 1). The studies differed, however, in the time that the animals were exposed to the running stimulus—5 days in our case and 12 days in the Farioli-Vecchioli study. These different time courses mean that the stage of maturation of the running-induced cells would have been different, and we suggested in our manuscript (Fischer et al., 2014), that the changes observed by Farioli-Vecchioli et al. may have been due to an increased proportion of their NeuroD1-labeled cells being late-stage precursors which are reported to have a shorter cell cycle length than the early type-2a cells (Brandt et al., 2012; see Table 1). The 12 days of exercise in their experiment would allow time for an increased bolus of new precursors to mature to faster-cycling type-3 cells leading to a shorter mean cell cycle length. In our study with the shorter 5-day paradigm, in contrast, fewer of the labeled cells would be expected to have advanced to the type-3 stage. That such apparently minor details in experimental methodology might lead to fundamentally different interpretations highlights the necessity for clear hypotheses to be generated to tease apart the complexity in this system.

**Table 1:** **Cell cycle length estimates in mouse hippocampal precursor cells**.

**Cell type**	**S**	**G2/M**	**G1**	**Total**	**References**
Mixed	8			16.1	Nowakowski et al., 1989
Mixed	7.6			12–14	Hayes and Nowakowski, 2002
Mixed	6			14	Burns and Kuan, 2005
Mixed	>7			14	Mandyam et al., 2007
Mixed	6.4	2.25	11.2	19.9	Beukelaers et al., 2011
Type-1	9.7	13.1		22.8	Brandt et al., 2012
Type-1/2a	13.5	13.5		27	Brandt et al., 2012
Type-2b/3	10.1	12.5		22.6	Brandt et al., 2012
Mixed	12.1	9	4.2	25.3	Brandt et al., 2012
Type-1	7.1			22.8	Farioli-Vecchioli et al., 2014
Type-2–3	9.9			22.3	Farioli-Vecchioli et al., 2014
Mixed	12.9	2.25	9.8	24.95	Farioli-Vecchioli et al., 2014
Mixed	11	~6.2	~5.5	22.7	Fischer et al., 2014
Type-1 (QNP)	7.8				Encinas et al., 2011
Type-1 (ANP)	12.2			28	Encinas et al., 2011

*Lengths of the separate stages of the cell cycle are given where available. ANP and QNP refer to the amplifying and quiescent neural progenitors defined by Encinas and colleagues*.

If, therefore, alteration of cell cycle length is not, as we argue, sufficient to explain the increase in numbers of proliferating cells seen after wheel running, then other mechanisms need to be proposed and tested.

## Alternative hypotheses

Several other hypotheses can be envisaged to explain the increase in proliferation after physical activity: (1) there may be a reduction in cell death of the proliferating cells; (2) the progenitor cells might undergo more cell divisions in exercising animals, thus delaying their exit from the cell cycle; (3) physical activity could lead to the increase of actively dividing progenitors by inducing quiescent stem cells to enter the cell cycle.

### Attenuation of cell death

One scenario which would alter the numbers of proliferating cells is if they were dying during the proliferative progenitor stages (type-1–3). It is known that most of the new born cells die before becoming mature neurons (Young et al., 1999; Biebl et al., 2000; Kempermann et al., 2003; Sun et al., 2004) and it has been suggested that this could occur during the progenitor stages (Sierra et al., 2010; Encinas et al., 2011). If the rate of cell death were altered by running so that more of the cells survived, then this could allow large increases in the number of proliferating cells. The work of Sierra and colleagues has shown that the number of cells positive for 5-bromo-2′-deoxyuridine (BrdU; another thymidine analog) doubles, as expected, over the first 24 h after a single dose of BrdU but then levels off before declining from the third day (Sierra et al., 2010). Other studies have reported a similar dynamic, describing a reduction in proliferating cells at time points before 3 days (Kronenberg et al., 2003; Mandyam et al., 2007), the earliest at which we might expect newly-born cells to exit the cell cycle. Other lines of evidence, however, suggest that neurogenic cells do not die until they reach the calretinin-expressing stage around 3–4 days after the initial division from a type-1 cell (Brandt et al., 2003; Kempermann et al., 2004). It has also been suggested that BrdU label dilution could be a factor in perceived cell loss even at time point earlier than 4 days (Dayer et al., 2003). Crucial to this discussion is the work with Bax knockout mice which are deficient in apoptosis (Knudson et al., 1995; Sun et al., 2004). Sun and colleagues showed that the numbers of proliferating cells did not differ between Bax knockout and wild-type mice, but the number of surviving post-mitotic cells increased dramatically—indicating that cell death exclusively occurs in the post-mitotic population (Sun et al., 2004). The interesting extension of this work to look at the proliferative response to wheel running in Bax knockout mice has not yet been performed. It nevertheless appears that if cell death were used as a regulatory mechanism in the control of proliferation after running, it would have to occur by a mechanism independent of Bax-mediated apoptosis. Recent work has indicated that just such a process, ferroptosis, might fit this profile (Gascón et al., 2016). Although regulation of cell death by physical activity remains an intriguing possibility, the discrepancies described above regarding the timing and which cell types are affected mean that more work is needed to comfortably incorporate this process into a global model of adult neurogenesis.

### Increased number of cell divisions

Increases in proliferating cell numbers do not necessarily imply reduced cell death of precursor cells, there could also be control of the rate of their exit from the cell cycle and advancement to the calretinin-expressing stage. Because calretinin reactivity does not appear in BrdU labeled cells until 1 day after BrdU labeling (Brandt et al., 2003), it would seem that the calretinin-expressing cells are daughters of a type-3 division. This means that an additional division in the progenitor lineage would delay exit from the cell cycle so that what would have been calretinin-positive cells, would now be proliferating type-3 cells. In fact, the opposite has been seen after 3 days of physical activity when an increased fraction of the doublecortin-positive cells were shown to have left the cell cycle and begin expressing calretinin (Brandt et al., 2010). Furthermore, this would imply, *ceteris paribus*, a doubling of the number of daughter cells in the affected stage. When mentally summing the cell numbers in these rough models, however, it should be borne in mind that a purely symmetrical mode of division has not been conclusively established for the progenitor populations and that the occurrence of asymmetric division would confuse predictions of cell numbers at each stage. Also, it is not certain exactly which stages are affected by physical activity. If an additional asymmetric division occurred in the early type-2a population, then the expected doubling in numbers would be diluted proportionately. It might also be possible that an additional cycle could occur at the type-1 stem cell phase as an extra neurogenic division in the Encinas model (Encinas et al., 2011). These questions can only be answered through **lineage tracing** experiments which follow the fate of single proliferating cells. To date, however, this technique has not been applied to investigate quantitative differences in cell division history in running vs. sedentary animals.

KEY CONCEPT 6. Lineage tracing This term refers to the following of the fate of a single cell during several rounds of division. This is typically achieved by sparsely labeling dividing cells of a known type and later identifying the remaining progeny. Lineage tracing provides detail that is lost in the study of a mixed population.

### Recruitment of quiescent stem cells

Another hypothesis, for which evidence has been accumulating recently, is that physical activity might induce the recruitment of rapidly dividing precursor cells from a quiescent stem cell pool. Several studies have presented evidence for the existence of subpopulations of type-1 stem cells with either quiescent or actively proliferating properties (Lugert et al., 2010; Bonaguidi et al., 2011; Shin et al., 2015; Gebara et al., 2016). In comparison to the number of actively dividing progenitor cells, the pool of stem cells that are quiescent and possess the potential to be activated is large (Lugert et al., 2010; Bonaguidi et al., 2011).

Central to the recruitment model is whether the type-1 cells increase division after physical activity and what the identity of their progeny might be. Several studies have reported that the number of cycling type-1 cells increases after running, although others suggest that this is not the case. Previous studies have suggested that type-1 cells do not respond to physical activity as after 4 weeks of running no increase in BrdU-labeled type-1 cells (based on Nestin-GFP^+^/GFAP^+^ phenotype, 24 h after BrdU injection) was observed (Kronenberg et al., 2003). Similarly no increase in numbers of radial type-1 cells after running (using Nestin-GFP mice) was reported in another study (Steiner et al., 2008). Other studies, however, have reported increases in type-1 cell proliferation after physical activity. Although running did not significantly change the total number of cells expressing Hes5-GFP^+^ (a marker of type-1 cells), the number of dividing Hes5^+^ cells (Hes5^+^/PCNA^+^) was increased by 12 days of running (Lugert et al., 2010). This increase was restricted to the radial rather than the horizontal Hes5^+^PCNA^+^ cells indicating that running recruits quiescent radial stem cells specifically without affecting the horizontal stem cells. The activated radial cells undergo asymmetric divisions (Bonaguidi et al., 2011; Encinas et al., 2011; Gebara et al., 2016) and thus total Hes5^+^ cell counts remained unchanged. Another report (Gebara et al., 2016) identified two classes of radial glia-like cell which they termed α cells, the predominant type which are proliferative and give rise to neurons and astrocytes; and β cells, which are non-proliferative, have a shorter and more highly branched process and may be astrocyte precursors. Running was shown to increase the number of α cells (Gebara et al., 2016).

The discrepancy as to whether the number of type-1 cells increases following physical activity may relate to the subpopulations of stem cells that were investigated in each study. Work on primary hippocampal cells *in vitro* has also reached the conclusion that at least two subpopulations of precursor cells exist, each with different properties regarding their ability to be activated (such as by KCl depolarization or by norepinephrine; Walker et al., 2008; Jhaveri et al., 2010, 2015). In this context, it cannot be excluded that wheel running presents a stimulus distinct from the baseline proliferation/recruitment in sedentary animals.

An alternative hypothesis might be that not only type-1 cells but also type-2 (and possibly even type-3) cells have the ability to enter a quiescent state in order to facilitate a prompt neurogenic reaction to environmental/behavioral changes (Suh et al., 2007). Whether these quiescent progenitors would undergo only symmetric division or have some limited capacity for self-renewal is still not clear.

## Open questions

As can be seen from this discussion, many open questions remain. Some key pieces of data will be required before a complete model can be constructed.

### Stage-specific quantification

Firstly, quantification of the number of cells at each different stage is necessary. Some attempts have been made (Kronenberg et al., 2003; Mandyam et al., 2007; Aelvoet et al., 2015) but this has not yet been done at acute time points over the first few days of running. Even the data that do exist are difficult to interpret as the numbers of cells at each stage do not follow the progression over time that would be predicted from the standard models.

### Cell cycle dynamics

A key factor in the confusion is that the neurogenic cells in the hippocampus are not synchronized, so that measures of proliferation yield superimposed results from cells at many different stages. This problem could be approached by cell stage-specific marker constructs for lineage tracing, especially if these were inducible, allowing a cohort of cells of a particular age to be followed as they mature. Such tools do not yet exist however. Lineage tracing has been performed to follow type-1 clones through multiple cell divisions (Bonaguidi et al., 2011; Encinas et al., 2011; Gebara et al., 2016), but not yet in the context of the effect of physical activity. There is also still no consensus on how many divisions are involved from type-1 progeny to the calretinin stage—indeed, the number of divisions may be variable. The ability to target studies at particular cell stages will also require the identification of new markers, ideally single proteins specific for each stage. Currently, researchers are limited either to combinations of marker proteins which limits the design of stage-specific expression vectors, or to single markers with broad expression profiles, such as nestin or NeuroD1, which do not allow the definition of unique stages without the addition of morphological criteria. The discovery of unique stage-specific markers, if these indeed exist, will be an important breakthrough for the field.

### Completeness of the underlying model

The sequence of stages, type-1–3 and beyond, is also not written in stone. Experiments focusing on individual cells *in vivo* have revealed that, at least at the stage of radial-glia-like precursor cells, there is a flexibility in fate (Bonaguidi et al., 2011; Sun et al., 2015; Gebara et al., 2016). Exercise also induces cell cycle exit (Brandt et al., 2010), and shortcuts to differentiation, such as from type-2a to post-mitotic maturation, might even be possible. The consequence is that the entire developmental backbone onto which the exercise stimulus acts appears to be very malleable. There is also the theoretical possibility that some cells expressing precursor cell markers might directly convert into neurons.

### Cell cycle length

There are also a few methodological discrepancies which need to be addressed. Firstly, as can be seen from Table 1, estimates of cell cycle length have not been consistent across different studies. A major difference is the distinction between the 14-h (Hayes and Nowakowski, 2002; Burns and Kuan, 2005; Mandyam et al., 2007) and 23-h (Cameron and McKay, 2001; Brandt et al., 2012; Farioli-Vecchioli et al., 2014; Fischer et al., 2014) total cell cycle lengths. It is not clear what is behind these differences in reported cell cycle lengths. Genetic differences are unlikely to be a cause as all studies were performed with the strain C57BL/6 except for one (Beukelaers et al., 2011) which used a mixed background. Indeed, Hayes and Nowakowski (2002) compared two strains with differing levels of proliferation and showed that the cell cycle dynamics were similar. It is possible that the cell cycle lengths might reflect the age of the experimental animals although, as ages were not always reported in these studies, it is not possible to comment further on this.

### Markers of proliferation

The choice of proliferation marker is also a significant factor affecting interpretation. The most widely used marker in the field is BrdU which, like the other thymidine analogs CldU and IdU, labels cells in S phase and their progeny. Thus, the number of cells marked by these compounds is not only dependent on the dosage and number of injections (defining how many dividing cells are exposed to the label) but also the time point after labeling at which the animals are killed (reflecting how many cell divisions can have occurred). For acute measures of proliferation, endogenous protein expression can yield more accurate results. The most popular of such markers, Ki67 (Gerdes et al., 1983, 1984), is expressed during most phases of the cell cycle and offers a robust estimate of the total number of actively dividing cells. On the other hand, the often-used marker PCNA has been shown to be astonishingly stable *in vivo*, labeling cells which divided up to 60 days previously (Mandyam et al., 2007) making this a poor choice for acute labeling, as results may be confounded by past cell cycle activity outside the scope of the experiment. Such technical vagaries mean that researchers may not always be measuring what they think they are and this could hinder correct interpretation of the results.

### Dynamics of wheel running

The amount of exposure to the running wheel also varies enormously between studies and makes synthesis of the literature difficult. The response of animals to the running stimulus depends on genetic background (Clark et al., 2011; Overall et al., 2013), group size (Stranahan et al., 2006; Leasure and Decker, 2009; Kannangara et al., 2011; Grégoire et al., 2014) and possibly also running wheel size and type. The reported distances run vary between studies (Lightfoot et al., 2010; Clark et al., 2011; Kohman et al., 2012; Overall et al., 2013) and may affect the rate of proliferation. It is also still unclear why the pro-proliferative effect of wheel running attenuates after longer bouts of activity (beyond 6 weeks) (Kronenberg et al., 2006; Overall et al., 2013). It may be possible that there exists a pool of progenitor cells exhibiting limited self-renewal which are responsible for the rapid response to the running stimulus but which require a longer period to be restocked from the stem cell source. It is unknown exactly what the identity of these cells would be however.

In summary, there are a number of possible mechanisms to explain the observation of increased precursor cell proliferation after physical activity but the evidence for and against each of these often appears contradictory. It seems likely that as yet undiscovered sources of complexity are hidden in what might at first appear to be a straightforward system, or perhaps we are hindered by misinterpretation of the data—attempting to place our results in the context of an insufficient theoretical framework.

## Implications of different models to an understanding of adult hippocampal neurogenesis

Despite being the subject of intense research for roughly 15 years now, the mechanisms regulating adult hippocampal neurogenesis are still not well understood. Many potential influences on precursor cell proliferation have been described (see discussion in Kempermann, 2011) and a large number of contributing molecular factors, onto which such influences might converge, have been identified (Overall et al., 2012). Nevertheless, a unifying model has not yet been proposed. For any such model to exist, it will be necessary to describe the dynamics of cell division for all the contributing precursor cell types and stages. It is not only frustrating to attempt to reconcile the existing data in the absence of a framework, but the lack of a coherent model also makes it more difficult to design future experiments.

Whether regulation of precursor cell proliferation occurs at the level of stem cells or in the type-2 progenitors will make a fundamental difference to strategies for manipulating it in research or therapy. Likewise, whether the mechanism of proliferation is one of cell cycle control, cell death or fate decision will alter dramatically our approaches to intervention.

It should also be remembered that regulation of adult neurogenesis by physical activity does not stand in isolation. We have argued before that the experimental model of wheel running is also a reflection of movement in the wild, and that locomotion and cognitive challenges are not completely independent (Kempermann, 2012, 2002). Given that adult hippocampal neurogenesis allows the flexible integration of novel information into existing contexts (Garthe et al., 2009), active exploration of the world, of which physical activity is only a part, is tightly linked to cognition and, hence, presumably even to adaptive success. Before that backdrop, the implications of a good model of the regulatory forces that act upon the precursor cells and link behavior with cellular actions are more far reaching than is apparent at first sight.

Work in the field is progressing rapidly and new techniques such as single-cell RNA sequencing and ever more specific constructs for lineage tracing are enabling many of the open questions presented above to be addressed. It is hoped that we are not too far away from working quantitative models which could allow the hoard of information currently available to be integrated into a functional, and predictive, overview.

## Author contributions

All authors listed, have made substantial, direct and intellectual contribution to the work, and approved it for publication.

### Conflict of interest statement

The authors declare that the research was conducted in the absence of any commercial or financial relationships that could be construed as a potential conflict of interest.
